# Balloon-assisted bioprosthetic or native aortic scallop intentional laceration to prevent iatrogenic coronary artery obstruction with en face view for patients exhibiting severe calcified leaflet: a case report

**DOI:** 10.1093/ehjcr/ytae643

**Published:** 2024-12-06

**Authors:** Yuta Kobayashi, Yusuke Enta, Masaki Nakashima, Norio Tada

**Affiliations:** Department of Cardiology, Sendai Kousei Hospital, 1-20, Tsutsumidori Amamiya-cho, Aoba-ku, Sendai, Miyagi 981-0914, Japan; Department of Cardiovascular Medicine, Faculty of Medicine and Graduate School of Medicine, Hokkaido University, Sapporo 060-8638, Japan; Department of Cardiology, Sendai Kousei Hospital, 1-20, Tsutsumidori Amamiya-cho, Aoba-ku, Sendai, Miyagi 981-0914, Japan; Department of Cardiology, Sendai Kousei Hospital, 1-20, Tsutsumidori Amamiya-cho, Aoba-ku, Sendai, Miyagi 981-0914, Japan; Department of Cardiology, Sendai Kousei Hospital, 1-20, Tsutsumidori Amamiya-cho, Aoba-ku, Sendai, Miyagi 981-0914, Japan

**Keywords:** BA-BASILICA, ViV-TAVI, Calcified leaflets, En face view, Case report

## Abstract

**Background:**

Balloon-assisted bioprosthetic or native aortic scallop intentional laceration to prevent iatrogenic coronary artery obstruction (BA-BASILICA) enables valve-in-valve transcatheter aortic valve implantation (ViV-TAVI) in patients at risk of coronary artery obstruction. However, its efficacy in patients with severely calcified leaflets remains unclear.

**Case summary:**

We report a 78-year-old woman with a deteriorated 21 mm Carpentier-Edwards PERIMOUNT Magna valve. Computed tomography showed severe calcification in the left coronary leaflet, extending above the left coronary artery (LCA) ostium, with a virtual transcatheter heart valve to coronary ostium distance of 3.7 mm, indicating a high risk of coronary obstruction after ViV-TAVI. We performed ViV-TAVI using the BA-BASILICA because of the patient’s high surgical risks. Traversal of the calcified leaflet was successfully achieved using both en face and side views to visualize the traversal system’s position in an area without calcification and in front of the LCA. After traversal, the leaflet was dilated with a balloon and accidentally split into two. A 20 mm SAPIEN 3 Ultra RESILIA valve was deployed. Despite initial procedural success, severe LCA stenosis developed due to leaflet compression. This was resolved by orthotopic stenting using an en face view to identify cells not covered by the bioprosthetic leaflet generated by BA-BASILICA.

**Discussion:**

To our knowledge, this is the first report of ViV-TAVI using the BA-BASILICA with an en face view of severely calcified leaflets. This case suggests that BA-BASILICA with an en face view could be effective for patients at high risk of coronary obstruction with severely calcified leaflets.

**Learning points**

- Valve-in-valve transcatheter aortic valve implantation (ViV-TAVI) using the balloon-assisted bioprosthetic or native aortic scallop intentional laceration to prevent iatrogenic coronary artery obstruction (BA-BASILICA) remains challenging for patients with severely calcified leaflets.
- We present a successful case of BA-BASILICA and orthotopic stenting following ViV-TAVI using en face view.
- This case report underscores the usefulness of the enface view in ViV-TAVI using BA-BASILICA.

## Introduction

In valve-in-valve transcatheter aortic valve implantation (ViV-TAVI), coronary artery obstruction occurs when the outwardly displaced leaflet blocks blood flow to the coronary artery ostium after the transcatheter heart valve (THV) is implanted.^1^ The balloon-assisted bioprosthetic or native aortic scallop intentional laceration to prevent iatrogenic coronary artery obstruction (BA-BASILICA) is an innovative technique that prevents coronary artery obstruction by electrically splitting the leaflet to create an open cell in front of the coronary artery for patients with high risk of coronary artery obstruction.^2^ However, its application, in patients with severely calcified leaflets, remains a subject of clinical interest.^1^

## Summary figure

**Table:** 

Timeline	
2007	A 64-year-old female commenced haemodialysis for end-stage renal failure caused by IgA nephropathy.
2014	Seven years after starting haemodialysis, she underwent surgical aortic valve replacement with 21 mm Carpentier-Edwards PERIMOUNT MAGNA for symptomatic aortic stenosis.
January 2023	She was admitted to our hospital with a chief complainant of dyspnoea. The diagnosis was severe structural valve deterioration (left ventricular ejection fraction of 46%, an effective orifice area of 0.50 cm², and a mean pressure gradient of 53 mmHg). Since computed tomography showed severe calcification of the leaflets and anatomically high risk of coronary artery occlusion, the heart team decided to perform valve-in-valve transcatheter aortic valve implantation (ViV-TAVI) with balloon-assisted bioprosthetic or native aortic scallop intentional laceration to prevent iatrogenic coronary artery obstruction (BA-BASILICA). Despite the use of BA-BASILICA, severe coronary stenosis occurred, and then orthotopic stenting was performed as a bailout procedure. The post-procedural course was uneventful without any neurological sequelae.

## Case presentation

**Figure 1:**
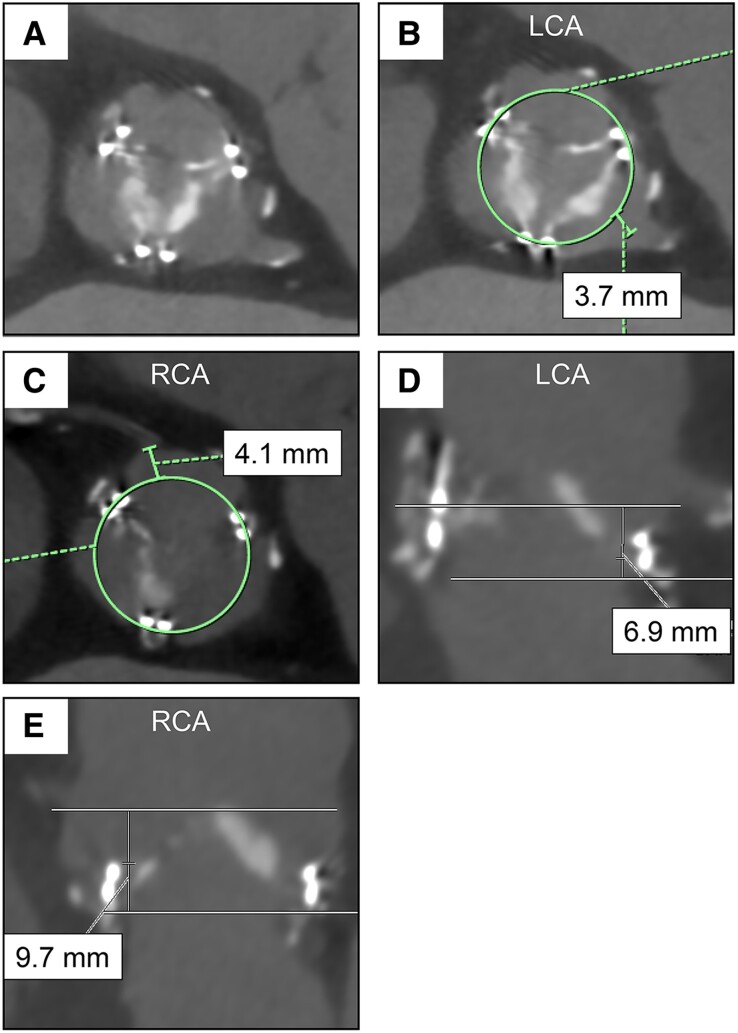
Preprocedural computed tomography. (*A*, *B*) Severely calcified and thickened leaflet. (*C*) Virtual transcatheter heart valve to left coronary ostium distance. (*D*) Virtual transcatheter heart valve to right coronary ostium distance. (*E*) Left coronary height. (*F*) Right coronary height. LCA, left coronary artery; RCA, right coronary artery.

A 78-year-old Asian female patient, who had been on dialysis for IgA nephropathy for the past 17 years, underwent aortic valve replacement with a 21 mm Carpentier-Edwards PERIMOUNT Magna valve 10 years ago. She was admitted to our hospital with the chief complaint of dyspnoea. Her vital signs were as follows: blood pressure, 108/74 mmHg; heart rate, 97 b.p.m.; and oxygen saturation, 96% on room air. A chest X-ray showed an enlarged cardiac silhouette, pulmonary congestion, and pleural effusion. Transthoracic echocardiography (TTE) showed a left ventricular ejection fraction (LVEF) of 46%, an effective orifice area of 0.50 cm², and a mean pressure gradient of 53 mmHg, indicating severe haemodynamic structural valve deterioration. Computed tomography revealed severe calcification in the left and non-coronary prosthetic valve leaflets (*Figure 1A*). Furthermore, the left coronary artery (LCA) and right coronary artery (RCA) ostium heights were of 6.9 and 9.7 mm, respectively, with no stenotic lesions in either artery. The virtual THV to the LCA ostium distance was 3.7 mm and to the RCA ostium was 4.1 mm, with the left coronary leaflet extending beyond the LCA ostium. These measurements indicated a high risk of LCA obstruction following ViV-TAVI (*Figure 1B–E*).^3^

Given the high surgical risk (Society of Thoracic Surgeons Predicted Risk of Mortality, 23.6%), we opted for transfemoral ViV-TAVI using the BA-BASILICA technique. As the right coronary cusp leaflet was less calcified and the virtual THV to the RCA ostium distance was >4 mm, we planned to perform BA-BASILICA only on the left coronary cusp (LCC) leaflet.

The procedure was performed under general anaesthesia and guided by transoesophageal echocardiography. We used a biplane angiographic suite with 8 × 8 inch flat-panel detectors to show an en face view of the aortic valve from a deep right anterior oblique cranial angle on the front panel and a side view from the left anterior oblique angle on the lateral panel.^4,5^

**Figure 2:**
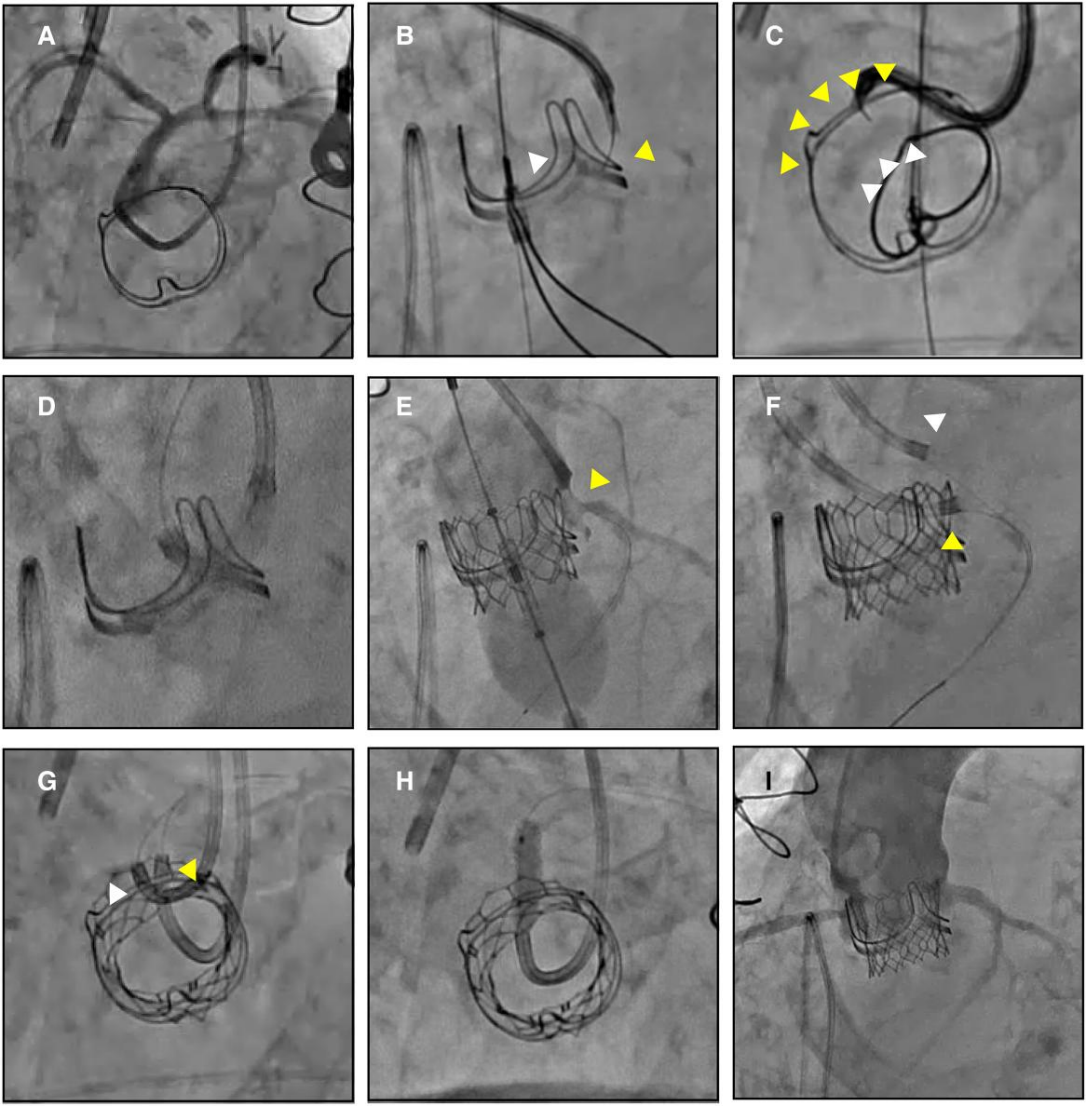
Intraprocedural angiography. (*A*) Coronary angiography on en face view. (*B*, *C*) Traversal on side and en face view. The en face view visualized calcified area (yellow arrow) and calcification-free area (white arrow). (*D*) Non-compliant balloon dilation of the left coronary leaflet. (*E*) Left coronary artery stenosis due to compression from the thickened leaflet. (*F–H*) Re-wiring and orthotopic stenting through SAPIEN3 Ultra RESILIA cell. Re-routed wire (yellow allow) and coronary protection wire (white arrow). (*I*) Patent flow to the left coronary artery after stenting.

Initially, we performed en face coronary angiography to confirm the orientation of the LCA along the short axis (*Figure 2A*). After confirming the distribution of LCC leaflet calcification and the position of the traversal system in an area without calcification and in front of the LCA using the en face view and verifying the optimal direction of the traversal system towards the LCC with a side view, a 0.014 inch, 300 cm Astato XS 9-40 (ASAHI-INTECC) wire was electrified in pure cut mode at 100 W while being gently advanced (*Figure 2B* and *C*; Supplementary material online, *Videos S1* and *S2*). The wire successfully passed the LCC, was snared within the left ventricle, and was externalized.

Next, according to the BA-BASILICA concept, the left leaflet was dilated using a 5 mm non-compliant balloon. During balloon expansion, both ends of the V-shaped wire were pulled to prevent positional shifting. However, this resulted in accidental splitting into two halves without electrosurgical laceration (*Figure 2D*). The patient’s haemodynamics remained stable.

**Figure 3:**
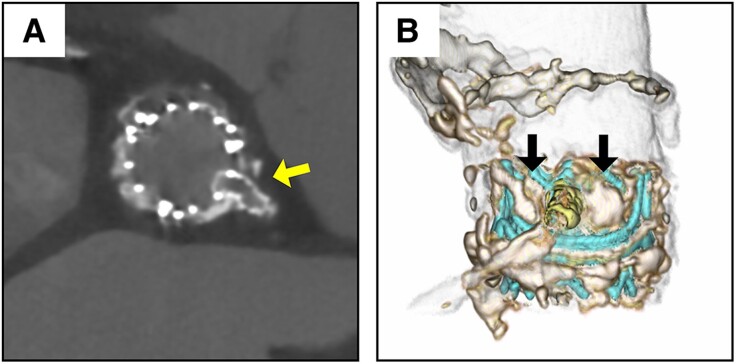
Post-procedural computed tomography. (*A*, *B*) Orthotopic stent (yellow arrow) and split leaflet (black arrow).

Despite the successful BA-BASILICA procedure, there was still a risk of coronary obstruction due to the avulsed thick leaflet and the short virtual THV to the LCA ostium distance. Therefore, we inserted a wire into the LCA and positioned a coronary artery balloon using a 7 Fr ASAHI Hyperion Judkins Left 3.5 catheter SH (ASAHI-INTECC) for coronary protection. We deployed a 20 mm SAPIEN 3 Ultra RESILIA (S3UR, Edwards Lifesciences, Irvine, CA, USA) in the nominal filling. However, the lacerated prosthetic valve leaflet compressed both the THV and the LCA ostium, resulting in suboptimal THV expansion, severe LCA stenosis, and the patient's haemodynamic collapse (*Figure 2E*; Supplementary material online, *Video S3*). To address this issue, a coronary artery balloon placed in the LCA was expanded to the coronary ostium, stabilizing the patient's haemodynamics. Next, in the en face view, the guidewire was re-routed to the LCA via the S3UR cell, which was not covered by the leaflet created by the traversal and laceration (*Figure 2F* and *G*). Orthotopic stenting was then performed, and a patent flow to the LCA was observed (*Figure 2H* and *I*; Supplementary material online, *Video S4*). The post-procedural course was uneventful, and no neurological sequelae were observed. Transthoracic echocardiography before discharge showed a LVEF of 45%, effective orifice area of 1.28 cm², mean pressure gradient of 14 mmHg, and mild paravalvular leakage (see Supplementary material online, *Video S1* and *Figure S1*). Computed tomography revealed an orthotopic stent positioned through an S3UR cell between the divided and calcified leaflets (*Figure 3A* and *B*).

At the 3-month follow-up visit, the patient was well. Repeat TTE showed a LVEF of 51%, effective orifice area of 1.25 cm², mean pressure gradient of 17 mmHg, and no paravalvular leakage.

## Discussion

Coronary obstruction, a potentially fatal complication during ViV-TAVI, has an incidence rate ranging from 2 to 3%, with an associated in-hospital mortality rate of ∼50%.^6,7^ The BASILICA technique is designed to prevent coronary obstruction by splitting the bioprosthetic aortic valve leaflet in front of the coronary artery using percutaneous electrosurgical techniques.^1^ Kitamura *et al*.^8^ demonstrated that the BASILICA technique significantly reduces the risk of coronary obstruction, lowering the incidence to just 4.6% among high-risk patients, more than 50% of whom were predicted to experienced coronary obstruction. The BA-BASILICA is a modification of the BASILICA technique, in which the leaflet is dilated with a non-compliant balloon prior to electrical laceration with a traversed wire.^2^ While the BA-BASILICA procedure is more complex than the BASILICA procedure, it has been reported to achieve a larger leaflet spray area than the BASILICA procedure. Notably, BA-BASILICA may be useful for calcified valves where BASILICA cannot achieve sufficient leaflet splay areas. However, the risk of coronary artery occlusion persists.^2^ This may be due to mechanical avulsion of the leaflet or the distance between the traversal point and the coronary ostium, causing the THV cell to be covered by the leaflet of the prosthetic valve.^2^ By using the en face view, it is possible to evaluate the positional relationship between the coronary ostium and the traversal site, allowing for the creation of a large space in front of the coronary ostium and a THV cell not covered by the leaflet of the prosthetic valve. This prevents coronary obstruction or orthotopic stenting.

Evaluating the traversal system position using both front and side views during traversal is recommended.^5^ Since the front and side views are both in the long-axis view, there are limitations in assessing the positional relationship between the traversal system and the coronary ostium and the calcification of the leaflet. In contrast, the biplane system with side and en face views provides a more detailed visualization of the traversal site. In the present case, the use of a strong penetration force traversal system (Astat XS 9-40 in 100 W pure cut mode), along with en face and side view biplane systems, allowed safe traversal in the area without calcification and in front of the LCA.

Recently, the effectiveness of SHORTCUT as a new leaflet modification device has been reported.^9^ It is expected to resolve the issues of procedural complexity and high difficulty associated with the BA-BASILICA procedure. The en face view is also considered to be beneficial when using SHORTCUT to evaluate the modification area and the position of the coronary arteries.

## Conclusion

Developing a strategy for TAVI in patients with calcified leaflets at risk of coronary artery occlusion remains a challenge. This case highlights the potential benefits of the BA-BASILICA technique in patients with high-risk coronary obstruction and severely calcified leaflets.

## Supplementary Material

ytae643_Supplementary_Data

## Data Availability

All data related to this case report are presented in the published manuscript.

## Lead author biography

Yuta Kobayashi, MD, PhD, graduated from Hirosaki University. He trained in cardiology at Sapporo City General Hospital, Tomakomai City Hospital, and Hokkaido University Hospital. Currently, he is undergoing structural heart disease intervention training in current institution.

## Notes

**Consent:** The authors confirm that written consent for submission and publication of this case report including images and associated texts has been obtained from all patients in line with the COPE guidance.

**Funding:** None declared.
