# Supplementary figures and images for: Balloon-assisted bioprosthetic or native aortic scallop intentional laceration to prevent iatrogenic coronary artery obstruction with en face view for patients exhibiting severe calcified leaflet: a case report
Source: Eur Heart J Case Rep. 2024 Dec 6;8(12):ytae643. doi: 10.1093/ehjcr/ytae643 (PMC11647591; doi:10.1093/ehjcr/ytae643)

## Slide 1
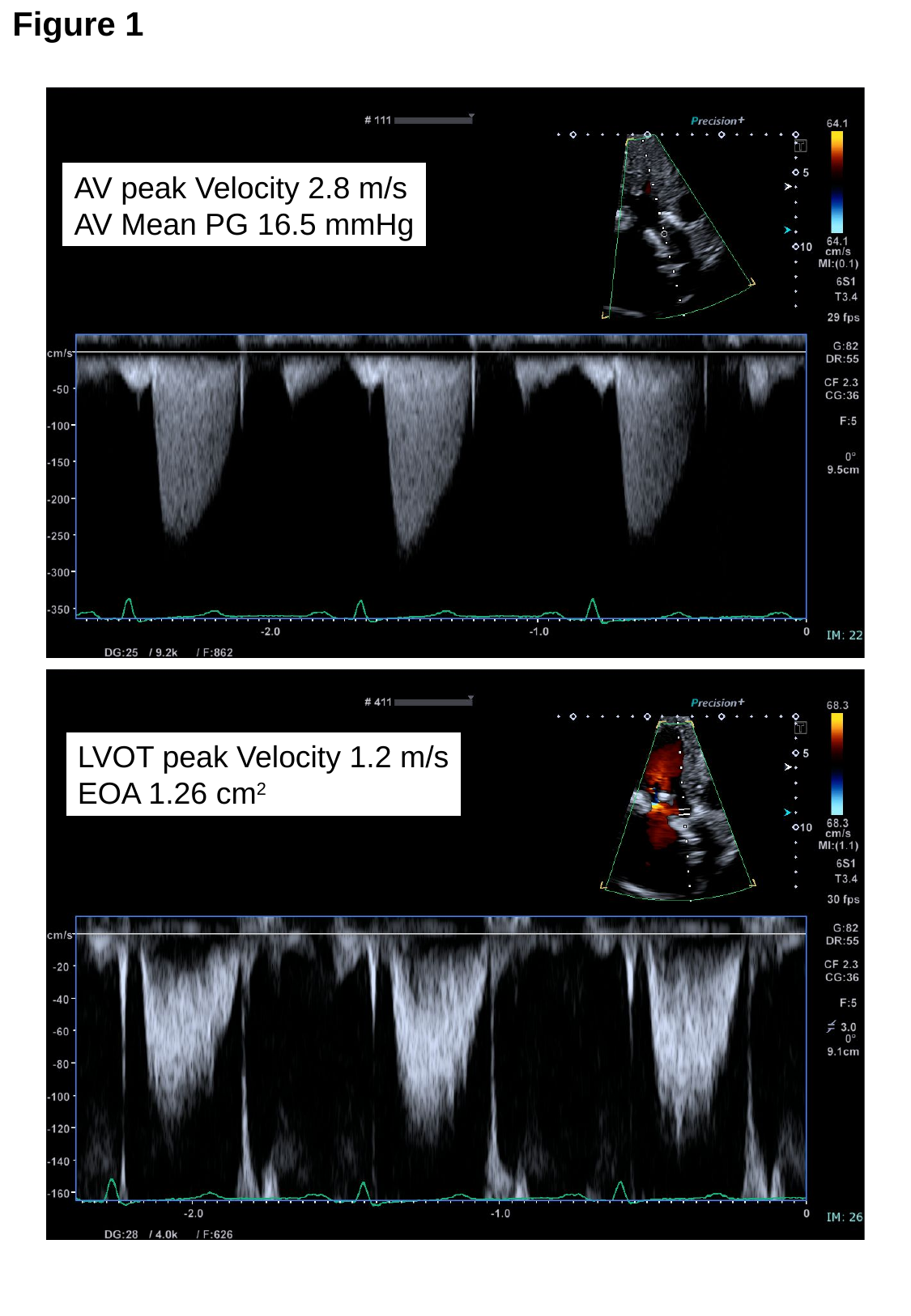

Figure 1
AV peak Velocity 2.8 m/s
AV Mean PG 16.5 mmHg
LVOT peak Velocity 1.2 m/s
EOA 1.26 cm2

Supplement: ytae643_Supplementary_Data [file ytae643_supplementary_data.zip › TAVI-BASILICA_S_Figures_YK.pptx]
